# Synthesis of NiCo_2_O_4_ Nanostructures and Their Electrochemial Properties for Glucose Detection

**DOI:** 10.3390/nano11010055

**Published:** 2020-12-28

**Authors:** Kyu-bong Jang, Kyoung Ryeol Park, Kang Min Kim, Soong-keun Hyun, Jae-eun Jeon, Young Sik Song, Soo-keun Park, Kyoung-il Moon, Chisung Ahn, Sung-chul Lim, Jaewoong Lee, Jong Cheol Kim, HyukSu Han, Sungwook Mhin

**Affiliations:** 1 School of Materials Science and Engineering, Inha University, 25 Younghyun-Dong, Incheon 22201, Korea; jkb0418@kitech.re.kr (K.-b.J.); skhyun@inha.ac.kr (S.-k.H.); 2 Department of Materials Science and Engineering, Hanyang University, 222 Wangsimni-ro, Seoul 04763, Korea; nebula9938@kitech.re.kr (K.R.P.); jaeeun00@kitech.re.kr (J.-e.J.); 3 Korea Institute of Industrial Technology, 137-41 Gwahakdanji-ro, Gangneung 25440, Korea; kmkim@kitech.re.kr; 4 Korea Institute of Industrial Technology, 156 Gaetbeol-ro, Incheon 21999, Korea; yssong@kitech.re.kr (Y.S.S.); pskeun@kitech.re.kr (S.-k.P.); kimoon@kitech.re.kr (K.-i.M.); cahn@kitech.re.kr (C.A.); lsc2001@kitech.re.kr (S.-c.L.); woong428@kitech.re.kr (J.L.); 5 Daegu Mechatronics & Materials Institute, Seongseogongdan-r0 11-gil, Dalseo-gu, Daegu 42714, Korea; 6 Department of Energy Engineering, Konkuk University, 120 Neungdong-ro, Seoul 05029, Korea; 7 Department of Advanced Materials Engineering, Kyonggi University, 154-42 Gwanggyosan-ro, Suwon 16227, Korea

**Keywords:** nickel cobaltite, enzyme-free, glucose sensor

## Abstract

In this work, we prepared spinel-type NiCo_2_O_4_ (NCO) nanopowders as a low-cost and sensitive electrochemical sensor for nonenzymatic glucose detection. A facile and simple chemical bath method to synthesize the NCO nanopowders is demonstrated. The effect of pH and annealing temperature on the formation mechanism of NCO nanoparticles was systematically investigated. Our studies show that different pHs of the precursor solution during synthesis result in different intermediate phases and relating chemical reactions for the formation of NCO nanoparticles. Different morphologies of the NCO depending on pHs are also discussed based on the mechanism of growth. Electrochemical performance of the prepared NCO was characterized towards glucose, which reveals that sensitivity and selectivity of the NCO are significantly related with the final microstructure combined with constituent species with multiple oxidation states in the spinel structure.

## 1. Introduction

Diabetes is a disease that impairs the human body’s ability to control glucose or sugar in the blood [1,2,3]. High glucose levels can cause serious health problems including heart disease, stroke, and kidney disease, and thus, it is important to maintain the glucose levels in blood via continuous monitoring with accurate detection of glucose [4,5,6]. Since the oxygen electrode was developed, different types of glucose sensors including an optical sensor and electrochemical sensor have been introduced [7,8]. Especially, the electrochemical glucose sensor has been actively studied to increase the sensitivity and to reduce the detection time for its excellent applicability to the real-time monitoring system.

Electrochemical glucose sensors can be divided into enzymatic and nonenzymatic sensors. The enzymatic glucose sensor exhibits high sensitivity and selectivity through direct immobilization of glucose oxidase. However, its natural limitations include a short lifetime due to poor chemical and thermal stability, and expensive processing costs hindering further advancement of the enzymatic biosensor for industrial applications [9,10,11]. Thanks to their several advantages such as long-term stability and reproducibility, and effective processing cost, different types of nonenzymatic electrocatalysts have been developed including noble metals, transition metals and alloys, and metal oxides [12,13,14]. Among them, transition-metal-based oxides have drawn great interest due to their configurational flexibility of transition metals, which promotes glucose oxidation with excellent sensitivity and selectivity [15,16,17,18].

Different types of synthesis methods for transition-metal-based oxides have been developed including hydroxide decomposition [19], nanocasting [20], electrodeposition [21], coprecipitation [22], and hydrothermal synthesis [23]. However, their complex and energy-consuming processing hinders the industrial application to glucose sensors. Therefore, exploring fast, environmentally friendly, and energy-efficient synthetic methods for transition-metal-based oxides is urgent. Recently, it has been reported that chemical bath synthesis has proven to be capable of controlling the size and morphology of materials by control of reaction parameters, such as temperature, pH, and solvent concentration [23,24,25,26]. It is expected that the transition-metal-based oxides prepared by chemical bath synthesis can be applied as a glucose sensor. However, there is limited information available on the effect of the processing parameters of chemical bath synthesis on the electrochemical performance for glucose detection. 

In this work, spinel-type NiCo_2_O_4_ (NCO) are successfully synthesized by a simple and facile chemical bath method. The morphology of the NCOs depending on pH during synthesis was investigated, which is closely related to the growth mechanism. Also, the formation of the NCOs was investigated to show excellent electrochemical performance for glucose detection including sensitivity, selectivity, and low detection limits.

## 2. Materials and Methods

### 2.1. Materials and Reagents

Nickel nitrate hexahydrate (Ni(NO_3_)_2_·6H_2_O, 99.99%), cobalt nitrate hexahydrate Co(NO_3_)_2_·6H_2_O, 99.9%), ammonia solution (NH_4_OH), sodium hydroxide (NaOH), D-(+)-glucose, uric acid (UA), dopamine (DA), L-ascorbic acid (LA), and acetic acid (AA) were purchased from Sigma-Aldrich (Seoul, Korea). All of the reagents used were of analytical grade and used as received without further purification.

### 2.2. Chemical Bath Synthesis of NiCo-Layered Double Hydroxide and NiCo_2_O_4_

An aqueous solution of Ni(NO_3_)_2_·6H_2_O (0.005 M) and Co(NO_3_)_2_·6H_2_O (0.01 M) was prepared by dissolving the salts in 100 mL of deionized water (DI water) with vigorous stirring for 60 min. Ammonia solution was added to the precursor solution until each scheduled pH value (11, 12, 13, and 14) was reached, followed by heating at 80 °C on a hotplate for 14 h, resulting in a thick, viscous, dark greenish fluid. It is noted that the pH of the precursor solution without the addition of ammonia solution was 8. Obtained products prepared at pH 8, 11, 12, 13, 14 are denoted as NCO8B, NCO11B, NCO12B, NCO13B, and NCO14B, respectively. The fluid was filtered through filter paper several times with DI water and ethanol. Subsequently, the filtered materials were dried in air for 24 h followed by annealing in air for 4 h at different temperatures at 450 °C with a heating rate of 10 °C/min, which turned the material black in color. Final products prepared at pH 8, 11, 12, 13, 14 are denoted as NCO8, NCO11, NCO12, NCO13, and NCO14, respectively. Also, crystalline NiCo_2_O_4_ with spinel structure is abbreviated as NCO. For clarity, detailed information is presented in Table S1 in the Supporting Information.

### 2.3. Material Characterizations and Electrochemical Measurements

The morphologies of NCO were investigated by scanning electron microscopy (SEM, Nova NanoSEM 450, FEI, Portland, OR, USA). The X-ray diffraction (XRD) patterns were collected using a PANalytical X-ray diffractometer (Empyrean, PANanalytical, Almelo, Nederland) with Cu-Kα radiation (λ = 0.1548 nm). 

All the electrochemical measurements including cyclic voltammetry (CV) and chronoamperometry (CA) were performed on an IVIUMSTAT electrochemical analyzer (IVIUMSTAT, Ivium Technologies, Eindhoven, Netherland) using a three-electrode system in a 0.1 M aqueous NaOH solution at room temperature. A glassy carbon electrode (GCE), an Ag/AgCl electrode, and a platinum plate were used as the working electrode, reference electrode, and counter electrode, respectively. The samples (10 mg), ethanol (0.5 mL), and Nafion solution (30 μL) were mixed for the preparation of the working electrode. Subsequently, drop-casting of the dispersion on the GC electrode was performed followed by drying under ambient conditions overnight.

The CV response was recorded between 0 and 0.6 V at different scanning rates of 5–100 mV/s. To get the optimal potential of the CA response of the sample, glucose was added to the 0.1 M NaOH solution at various potentials from +0.4 to +0.6 V, as shown in Figure S1. The optimal potential of +0.5 V was chosen, which was highly responsive and stable as the working potential for glucose detection. The CA response of the samples to the glucose was carried out at an applied potential of 0.5 V under stirred conditions. For sensing performance evaluation, 0.01–6 mM glucose solutions were used, with LA, DA, AA, and UA detection performed at concentrations of 0.1 mM in 0.1 M NaOH alkaline electrolyte. 

## 3. Results

**Figure 1:**
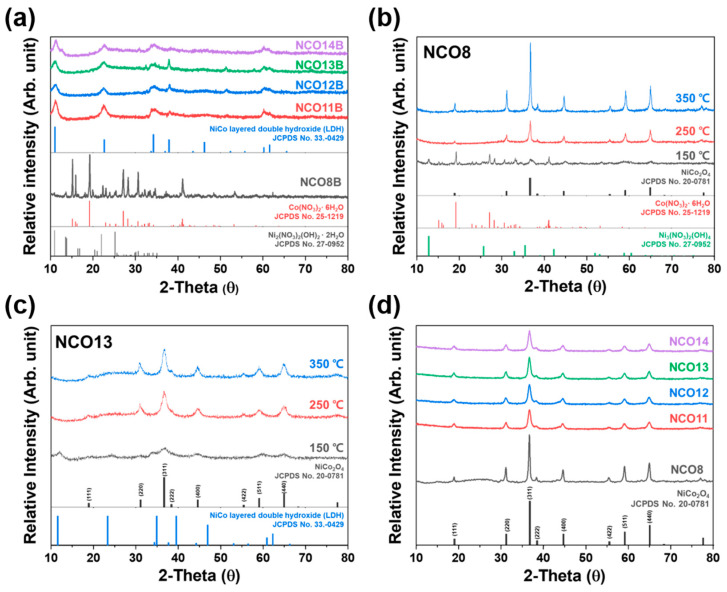
XRD patterns of (**a**) NCOBs (8B, 11B, 12B, 13B, and 14B), (**b**) NCO8 by different annealing temperatures (150, 250, and 350 °C), (**c**) NCO13 by different annealing temperatures (150, 250, and 350 °C), and (**d**) NCOs (8, 11, 12, 13, and 14) after annealing at 450 °C.

The influence of pH during synthesis on the crystallization of the NCO was investigated as shown in Figure 1. As shown in Figure 1a, Ni_2_(NO_3_)_2_(OH)_2_ · 2H_2_O and Co(NO_3_)_2_· 6H_2_O were observed from NCO8B, while NiCo-layered double hydroxide (NiCo-LDH) was observed from NCO11B to 14B [27]. As annealing temperature increased, different phase transformations were observed depending on pH as shown in Figure 1b, c. Phase transformation of the NCO8B to spinel-type NCO (NCO8) occurred from 150 °C, and NCO single phase with improved crystallinity was observed at 350 °C, which implies the chemical reaction of the intermediates (Ni_3_(NO_3_)_2_(OH)_4_ and Co(NO_3_)_2_· 6H_2_O) with oxygen for the formation of spinel-type NCO8. However, NCO11B–14B with LDH structure directly transformed into spinel-type NCOs (NCO11—14) without any chemical reaction between intermediates with increasing temperature. Regardless of pH during synthesis, spinel-type NCO single phase was observed after annealing at 450 °C (Figure 1d) [28]. In the chemical bath process, an increase of OH^−^ ions in a precursor solution containing Ni^2+^, Co^2+,^ and NO_3_^−^ occurred by applying NH_4_OH, which turned into an alkaline condition (pH = 11~14) in the chemical bath. Under the condition, the chemical reaction among different ions such as Ni^2+^, Co^2+^, and OH^−^ ions led to the formation of the NiCo-LDH as expressed by reaction (1) [29,30]. Simultaneously, H_2_O molecules and NO^3−^ ions were intercalated into NiCo-LDH interlayer to retain the LDH structure through a hydrogen bond. Subsequent annealing of the as-synthesized NiCo-LDH caused structural transformation into spinel-type NiCo_2_O_4_ (NCO), as described by the reaction (2) [29,30,31,32].

Ni^2+^ + 2Co^2+^ + 6OH^−^ → NiCo_2_(OH)_6_ (NiCo-LDH) (1)

2NiCo_2_(OH)_6_ + O_2_ → 2NiCo_2_O_4_ + 6H_2_O (2)

**Figure 2:**
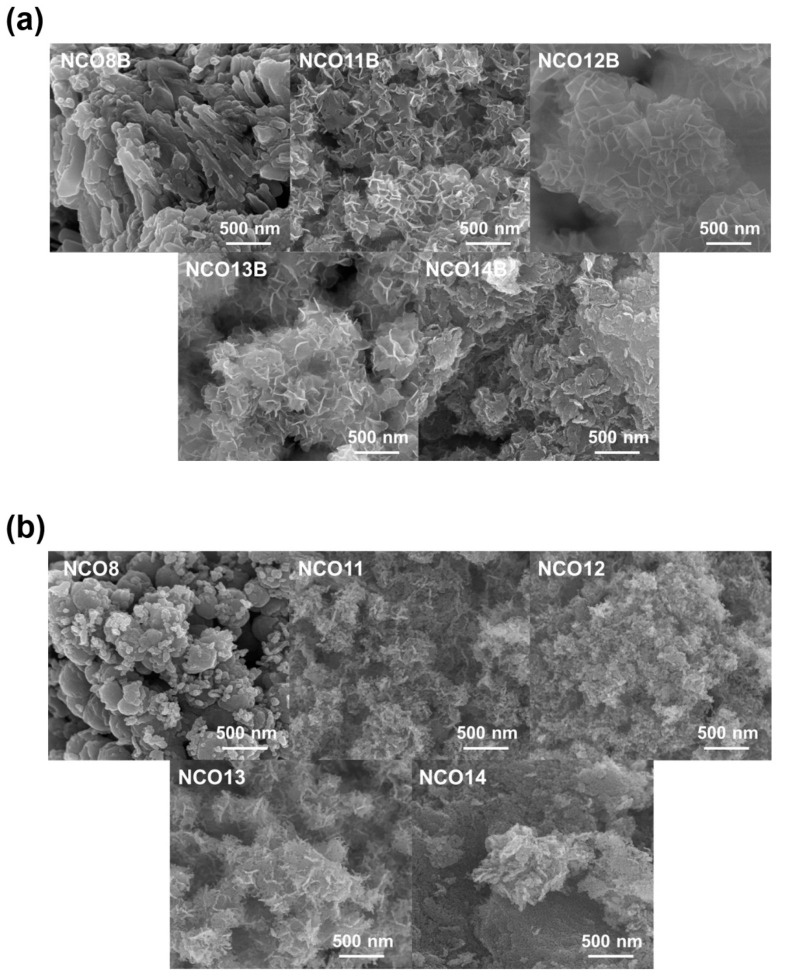
FE-SEM images of (**a**) NCOBs (8B, 11B, 12B, 13B, and 14B), and (**b**) NCOs (8, 11, 12, 13, and 14).

The pH depending on OH^−^ ions in the precursor solution also determines the morphology of the NCO during synthesis. As depicted in Figure 2a, a high concentration of OH^−^ ions for the reaction environment reveals the flower-like morphology as observed from NCO11B–13B, which is originated from anisotropic grain growth of LDH. [33,34] However, the unique flower-like morphology disappears at pH 14 (NCO14B) due to further grain growth of LDHs. After annealing, the morphological transformation of the NCOB prepared at different pH values was observed as shown in Figure 2b. Spherical nanoparticles were observed from NCO8, derived by the chemical reaction between nitrates and oxygen. However, the transformation from NiCo-LDH prepared at high pH (NCO11–13) to NCO maintains the sheet-like morphology. It is noted that the nanosheets consist of the assembly of spherical nanoparticles after transformation from LDH to NCO. Also, an increase of pH from 8 to 13 during synthesis results in smaller particle sizes after annealing. As expected, the morphology of the NCO14 is transferred from NCO14B. Regardless of different pH, homogenous distribution of the constituent elements (Ni, Co, and O) was observed from NCOBs and NCOs as shown in Figure S2, which implies that the synthesis route using the chemical bath method is applicable to prepare the spinel-type NiCo_2_O_4_ nanostructures at comparatively low temperature (450 °C). 

**Figure 3:**
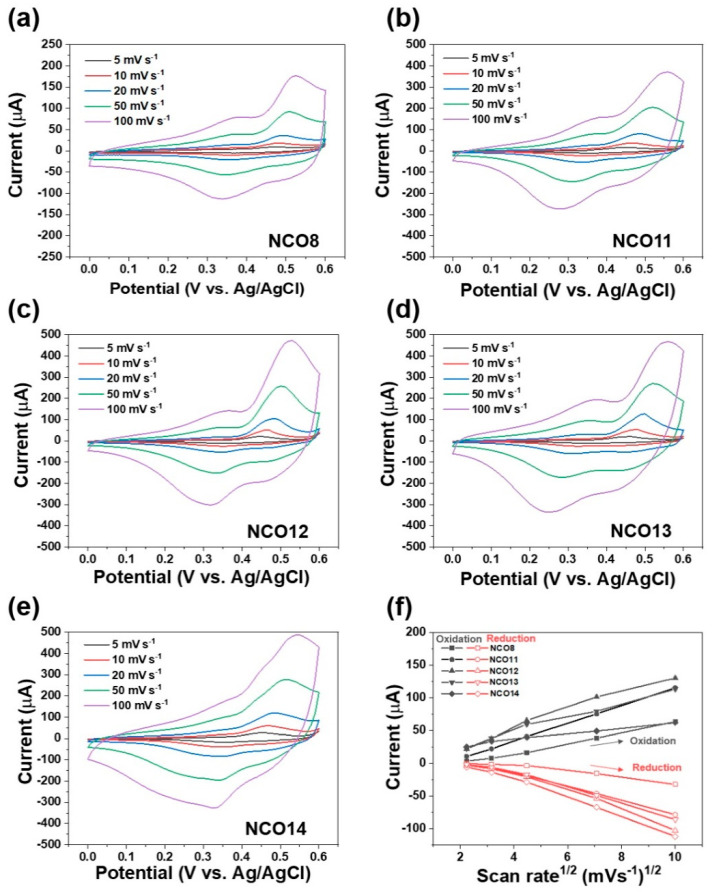
CV curves of (**a**) NCO8, (**b**) NCO11, (**c**) NCO12, (**d**) NCO13, and (**e**) NCO14 electrodes at different scan rates in 0.1 M NaOH solution. (**f**) Respective Randles–Sevcik plots of NCO electrodes.

Different microstructures of the NCOs can be determined by different processing conditions including pH, thus showing different electrochemical properties. The dependence of the cyclic voltammetric (CV) curves for NCOs on the pH during chemical bath synthesis was measured to investigate the electrochemical behavior of the NCOs under alkaline conditions (0.1 M NaOH) at various scan rates as shown in Figure 3. There is a negligible effect of pH on the redox peak potentials for the NCO electrodes. Regardless of pH, redox peak currents of the NCOs were increased with increasing CV scan rate. Oxidation peaks of the NCOs correspond to Ni^2+^/Ni^3+^, Co^2+^/Co^3+^, and Co^3+^/Co^4+^ due to oxidation of Ni^2+^, Co^2+^ and Co^3+^ to Ni^3+^, Co^3+^, and Co^4+^, respectively. It is noted that the redox peak potential of Co^3+^/Co^4+^ is close to that of Ni^2+^/Ni^3+^ and Co^2+^/Co^3+^, which shows overlapped redox peaks in the CV curve [35,36,37]. In addition, the reduction peaks of NCOs are attributed to the Ni^3+^, Co^4+^, and Co^3+^ to Ni^2+^, Co^3+^, and Co^2+^, respectively. The redox peak currents for the NCOs at the square root of the scan rates are presented in Figure 3f. All NCOs synthesized at different pH show a linear proportionality relationship between the redox peak currents and the square root of the scan rates, suggesting that NCOs undergo diffusion-controlled electrochemical behavior [38,39]. Also, CV responses of NCOs were synthesized at different pH in response to 5 mM glucose under alkaline conditions (0.1 M NaOH) at a scan rate of 50 mVs^−1^, as shown in Figure S3. Regardless of the pH, all NCOs oxidize glucose (C_6_H_12_O_6_) to gluconolactone (C_6_H_10_O_6_), which implies that NCOs synthesized at different pH can be applied to electrochemical glucose sensors [40,41].

**Figure 4:**
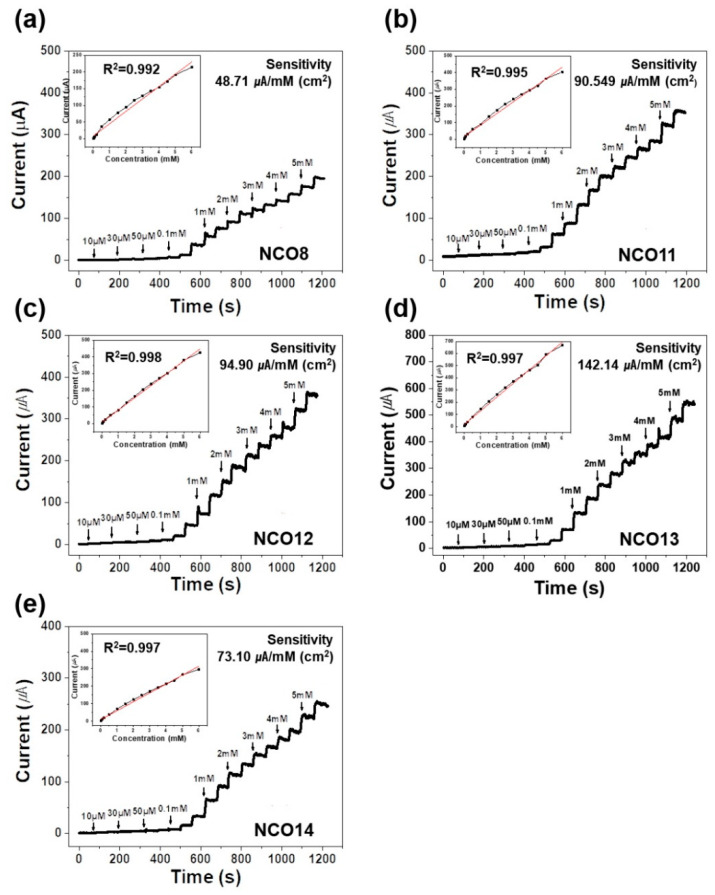
CA responses of (**a**) NCO8, (**b**) NCO11, (**c**) NCO12, (**d**) NCO13, and (**e**) NCO14 electrodes with the addition of glucose to 0.1 M NaOH solution at 0.50 V. Their respective calibration curves of current response versus glucose concentration plots inset in the figure.

The electrochemical performance of NCOs on glucose oxidation was investigated as shown in Figure 4. Chronoamperometry (CA) responses of NCOs were measured by stepwise changes in glucose concentrations in 0.1 M NaOH at 60 s intervals under an applied potential of 0.50 V. With increasing pH during synthesis, the sensitivity of NCO shows in the range between 48.71–146.26 μA/mM (cm^2^) with 0.995–0.998 (R^2^), which shows a linear detection limit in the range between 0.01–6 mM. The limits of detection (LOD) of NCOs are in the range between 0.0475–0.393 µM, as shown in Figure S4. Based on the results from the CA test, NCO13 shows superior electrochemical performance for glucose detection, supported by excellent linear sensitivity (146.24 μA/mM (cm^2^)) in a wide detection range. The excellent sensitivity of the NCOs is strongly associated with the redox reaction of active sites. As expected, Co^2+^, Co^3+^, Ni^2+^, and Ni^3+^ as active sites in NCO were investigated in XPS results, as shown in Figure S5. In the XPS spectra of Co2p and Ni2p, Co^2+^/^3+^ and Ni^2+^/^3+^ were observed on the NCO. It is believed that multi-valence states of Ni and Co cations play an important role as oxidizing agents for glucose detection [17,42,43]. Thus, the reversible conversion of Ni^2+^/Ni^3+^ and Co^2+^/Co^3+^ in NCOs enables repetitive glucose detection [44,45,46].

**Figure 5:**
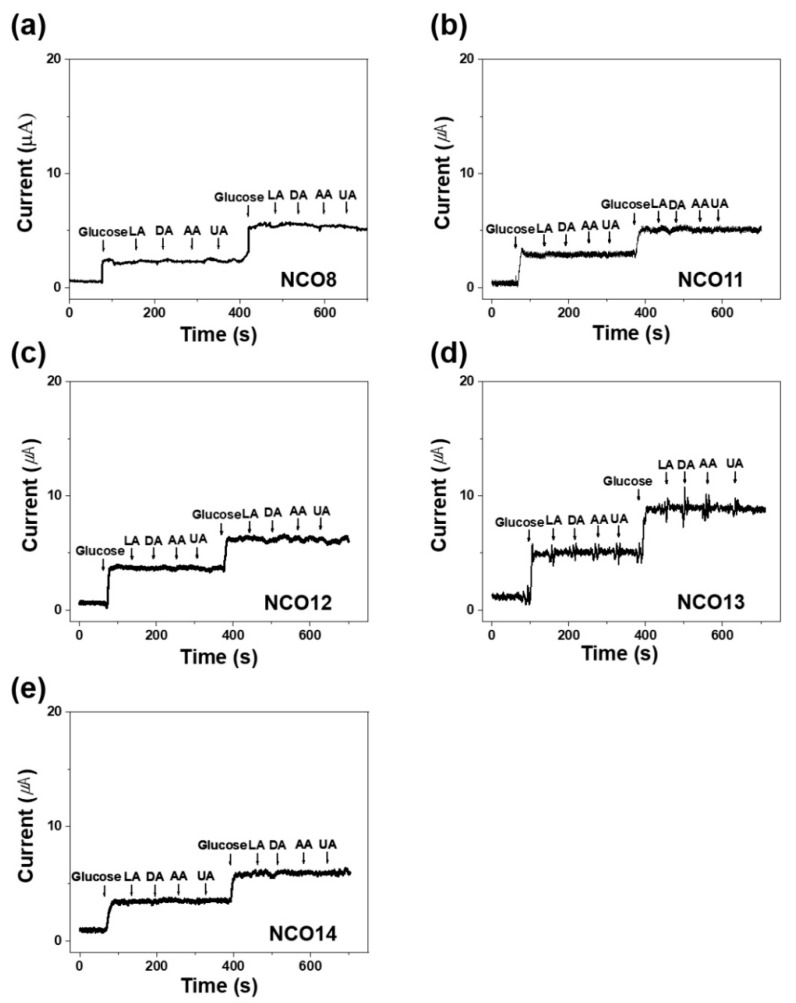
CA responses (CA) of (**a**) NCO8, (**b**) NCO11, (**c**) NCO12, (**d**) NCO13, and (**e**) NCO14 electrodes to addition of 1 mM glucose and 0.1 mM interfering species (LA, DA, AA, and UA) in a 0.1 M NaOH solution at 0.5 V.

The selectivity of NCOs is also an important factor for accurate glucose detection: current response to other reagents can detract from the determination of glucose [47,48,49]. The selectivity of the NCOs depending on different pH was investigated as shown in Figure 5. The current response of all NCOs to glucose is obvious. However, there is no change in the current response to uric acid (UA), dopamine (DA), L-ascorbic acid (LA), and acetic acid (AA) at the same glucose concentration of 1 and 2 mM, which implies that NCOs have excellent selectivity for glucose [41,50,51]. Based on the results above, it is suggested that the sensitivity of the NCOs for glucose detection is strongly dependent on morphology, however, selectivity for glucose detection is significantly determined by the redox reaction of the chemical components. The electrochemical performance of the NCOs prepared in this work was summarized in Table 1. The sensitivity of the NiCo_2_O_4_/rGO shows the highest value when compared to the other materials including NCOs, induced by the excellent electrochemical performance of the NCOs combined with fast electron transfer from supportive rGO [52,53,54,55]. However, NCOs show higher sensitivity with a lower detection limit in response to glucose compared to the rest of the materials in Table 1 [56,57,58]. Therefore, the NCOs as pure oxides can be expected to be practical for the application of glucose sensors.

## 4. Conclusions

Spinel-type NiCo_2_O_4_ (NCO) nanostructure was synthesized by a simple chemical bath method for electrochemical glucose sensors. Although different chemical reactions and formation of intermediates depending on pHs occur during synthesis, only spinel-type NCO was prepared after annealing at 450 °C. However, the morphology and particle size of the NCOs are strongly influenced by pH value, which emphasizes the importance of synthetic routes for the formation of the NCO. In our study, NCO13 with flower-like morphology assembled with small nanoparticles shows superior glucose detection including a sensitivity of 143 μA/mM (cm^2^) up to 6 mM with good linearity. It is revealed that the different morphology and particle size of the NCO determine the sensitivity for glucose detection. Also, the selectivity of the NCO is determined by the unique spinel structure and redox reaction of Ni and Co ions.

## Figures and Tables

**Table 1:** Comparison of our work to the nonenzymatic glucose sensor.

Electrode	Method	Sensitivity (μA/mM (cm^2^))	Linear Range (mM)	Correlation Coefficient (R^2^)	Detection Limit (μM)	Refs.
NCO8	CA	48.71	0.01–6	0.992	0.539	This work
NCO11	CA	90.549	0.01–6	0.995	0.393	This work
NCO12	CA	94.90	0.01–6	0.998	0.0503	This work
NCO13	CA	142.14	0.01–6	0.997	0.0433	This work
NCO14	CA	73.10	0.01–6	0.996	0.0475	This work
NiCo_2_O_4_/CNT	CA	66.15	0.02–12.12	0.99	5	[52]
NiCo_2_O_4_/rGO	CA	548.9	0.005–8.56	0.99	2	[53]
CuCo_2_O_4_	CA	3.625	Up to 0.32	-	5	[56]
NiO	CA	32.91	Up to 1.94		1.28	[57]
Co_3_O_4_	CA	36.25	Up to 2.04		0.97	[58]

## Supplementary Materials

The following are available online at https://www.mdpi.com/2079-4991/11/1/55/s1, Figure S1: CA response of NCO13 electrode upon addition of 1 mM glucose in 1m M NaOH solution at different applied potentials. Figure S2: SEM-elemental mapping images of (a) NCOBs (8B, 11B, 12B, 13B, and 14B), and (b) NCOs (8, 11, 12, 13, and 14). Figure S3: CV curves of (a) NCO8, (b) NCO11, (c) NCO12, (d) NCO13, and (e) NCO14 electrodes in the absence of glucose and with 5 mM concentration of glucose at a scan rate 50 mVs^−1^. Figure S4: CA response of (a) NCO8, (b) NCO11, (c) NCO12, (d) NCO13, and (e) NCO14 electrodes with the addition of 10 μM glucose in 0.1 M NaOH solution at 0.50 V. Figure S5: The XPS spectra of Ni2p and Co2p (NCO13). Table S1: Sample notations of As-prepared and after annealing samples.
