# Supplementary material for: Synthesis of NiCo2O4 Nanostructures and Their Electrochemial Properties for Glucose Detection
Source: Nanomaterials (Basel). 2020 Dec 28;11(1):55. doi: 10.3390/nano11010055 (PMC7824400; doi:10.3390/nano11010055)
Supplement: Supplementary file 1 [file nanomaterials-11-00055-s001.pdf]

nanomaterials

Supporting Information

Synthesis of $NiCo_2O_4$ Nanostructures and Their Electrochemial Properties for Glucose Detection

Kyu-bong Jang [1,†], Kyoung Ryeol Park [2,†], Kang Min Kim [3,†], Soong-keun Hyun [1], Jae-eun Jeon [2], Young Sik Song [4], Soo-keun Park [4], Kyoung-il Moon [4], Chisung Ahn [4], Sung-chul Lim [4], Jaewoong Lee [4], Jong Cheol Kim [5,*], HyukSu Han [6,*] and Sungwook Mhin [7,*]

[1] School of Materials Science and Engineering, Inha University, 25 Younghyun-Dong, Incheon 22201, Korea; jkb0418@kitech.re.kr (K.-b.J.); skhyun@inha.ac.kr (S.-k.H.)
[2] Department of Materials Science and Engineering, Hanyang University, 222 Wangsimni-ro, Seoul 04763, Korea; nebula9938@kitech.re.kr (K.R.P.); jaeeun00@kitech.re.kr (J.-e.J.)
[3] Korea Institute of Industrial Technology, 137-41 Gwahakdanji-ro, Gangneung 25440, Korea; kmkim@kitech.re.kr (K.M.K)
[4] Korea Institute of Industrial Technology, 156 Gaetbeol-ro, Incheon 21999, Korea; yssong@kitech.re.kr (Y.S.S.); pskeun@kitech.re.kr (S.-k.P.); kimoon@kitech.re.kr (K.-i.M.); cahn@kitech.re.kr (C.A.); lsc2001@kitech.re.kr (S.-c.L.); woong428@kitech.re.kr (J.L.)
[5] Daegu Mechatronics & Materials Institute, Seongseogongdan-r0 11-gil, Dalseo-gu, Daegu 42714, Korea
[6] Department of Energy Engineering, Konkuk University, 120 Neungdong-ro, Seoul 05029, Korea
[7] Department of Advanced Materials Engineering, Kyonggi University, 154-42 Gwanggyosan-ro, Suwon 16227, Korea
* Correspondence: jckim@dmi.re.kr (J.C.K.); hhan@konkuk.ac.kr (H.H.); swmhin@kgu.ac.kr (S.M.)
† These authors contributed equally to this work.

Table 1. Sample notations of As-prepared and after annealing samples.

Sample name	pH value				
	8	11	12	13	14
As-prepared (NCOBs)	NCO8B	NCO11B	NCO12B	NCO13B	NCO14B
Annealing at 450 °C (NCOs)	NCO8	NCO11	NCO12	NCO13	NCO14

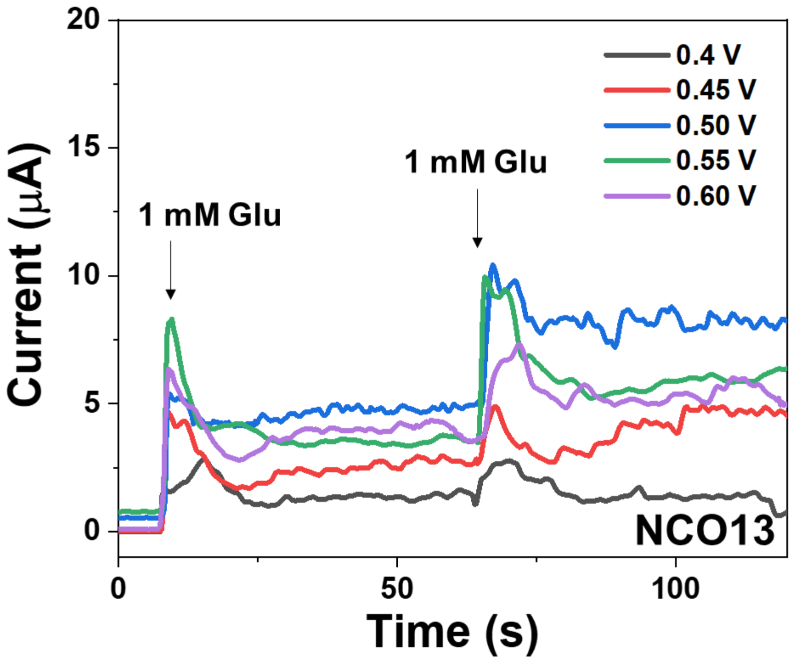

Figure S1. CA response of NCO13 electrode upon addition of 1 mM glucose in 1m M NaOH solution at different applied potentials.

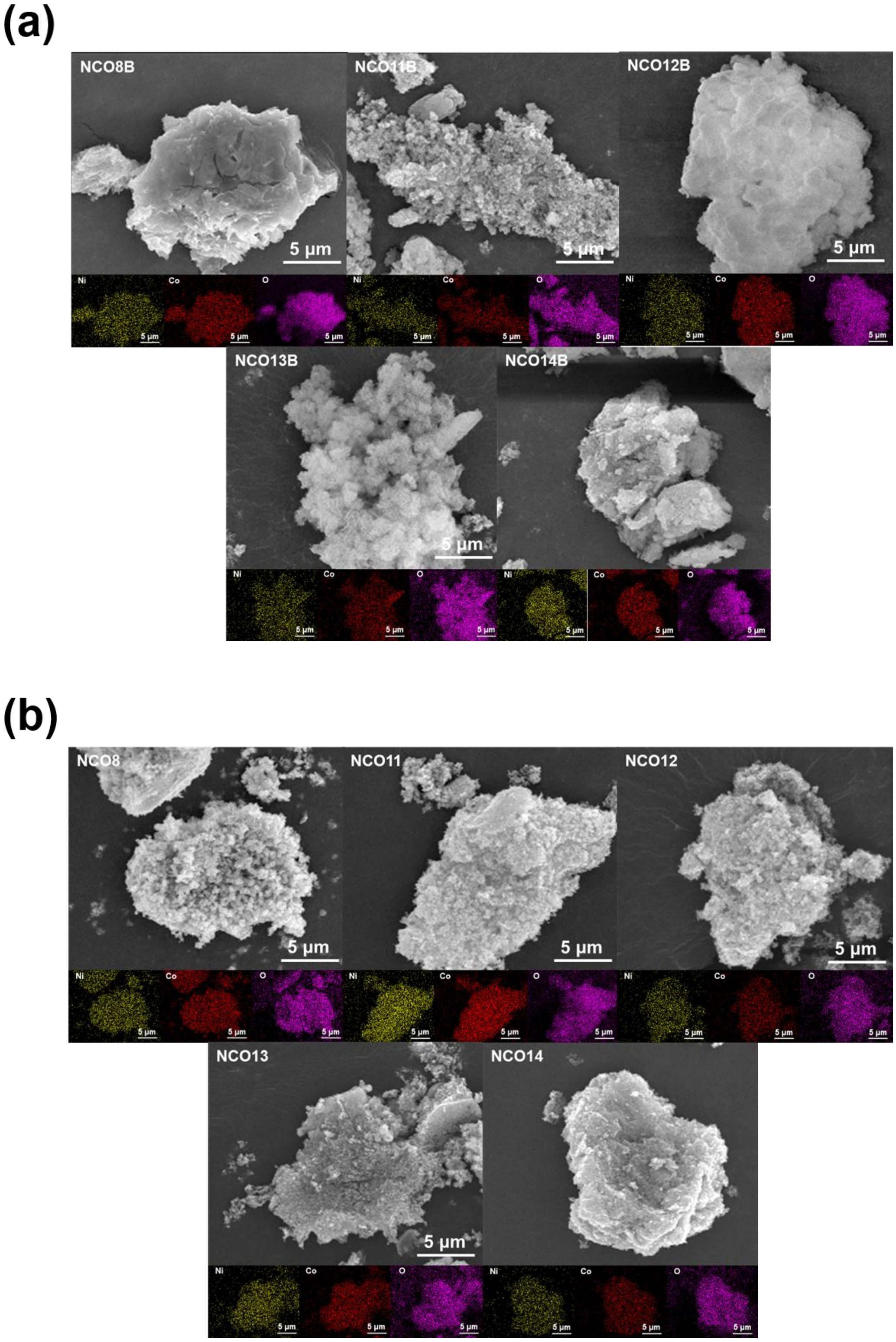

Figure S2. SEM-elemental mapping images of (**a**) NCOBs (8B, 11B, 12B, 13B, and 14B), and (**b**) NCOs (8, 11, 12, 13, and 14).

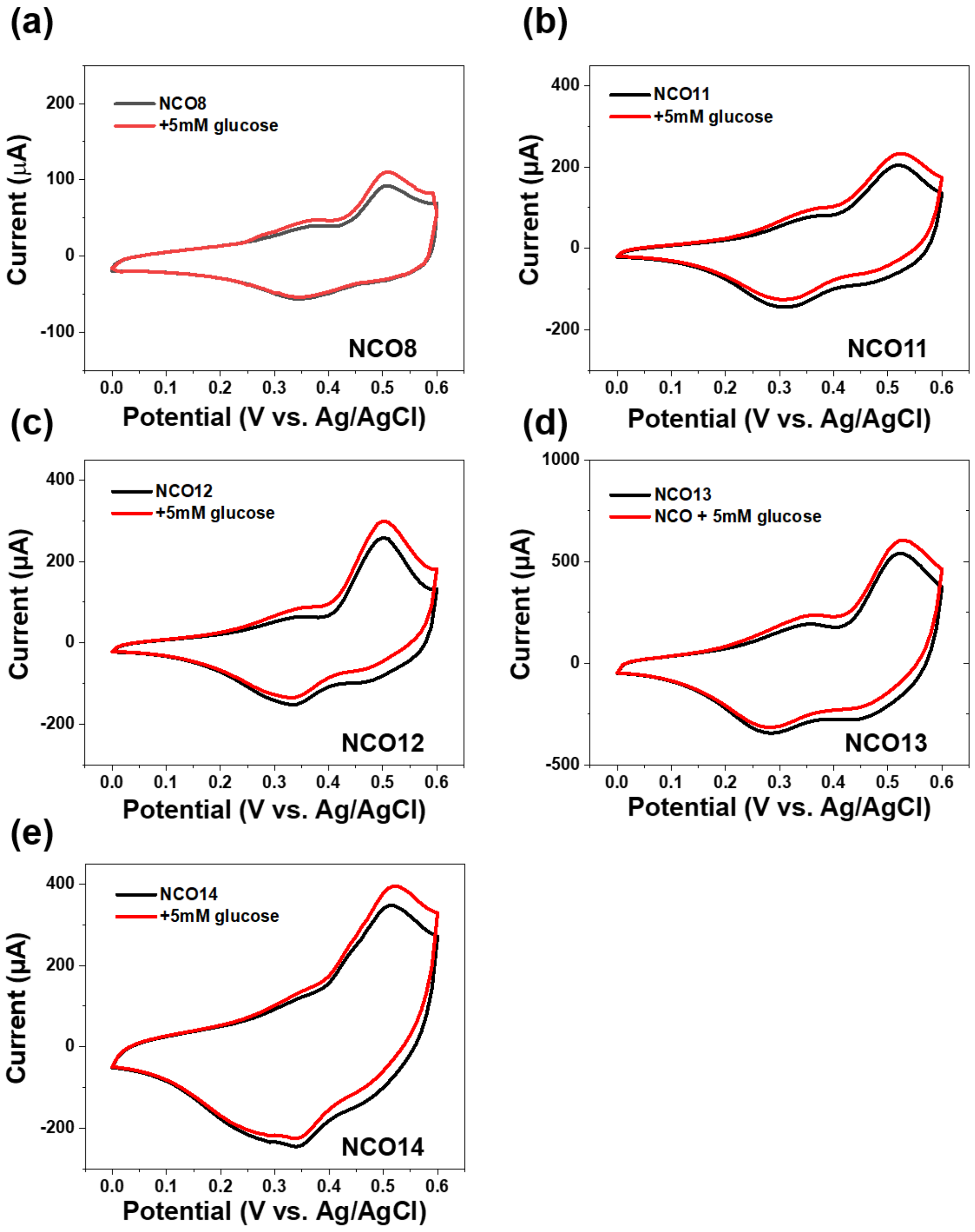

Figure S3. CV curves of (**a**) NCO8, (**b**) NCO11, (**c**) NCO12, (**d**) NCO13, and (**e**) NCO14 electrodes in the absence of glucose and with 5 mM concentration of glucose at a scan rate 50 mVs^{-1}.

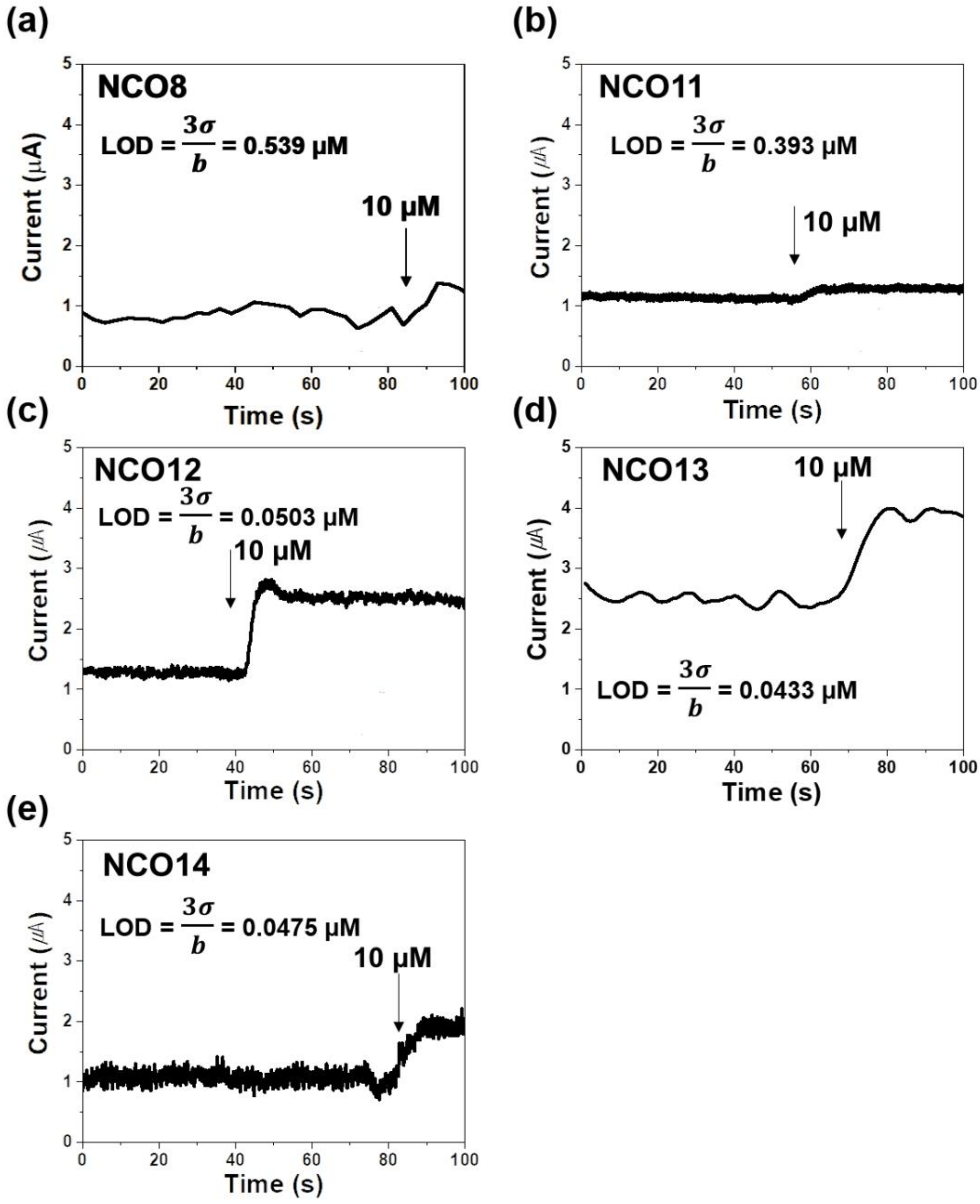

Figure S4. CA response of (**a**) NCO8, (**b**) NCO11, (**c**) NCO12, (**d**) NCO13, and (**e**) NCO14 electrodes with the addition of 10 μM glucose in 0.1 M NaOH solution at 0.50 V. The LOD (Limit of detection) calculated by the formula in term of LOD = $3\sigma/b$, where σ is the standard deviation of background which is obtained by measuring the current response of NCOs electrode in the 0.1M NaOH solution without glucose, and *b* is the sensitivity of the NCOs.

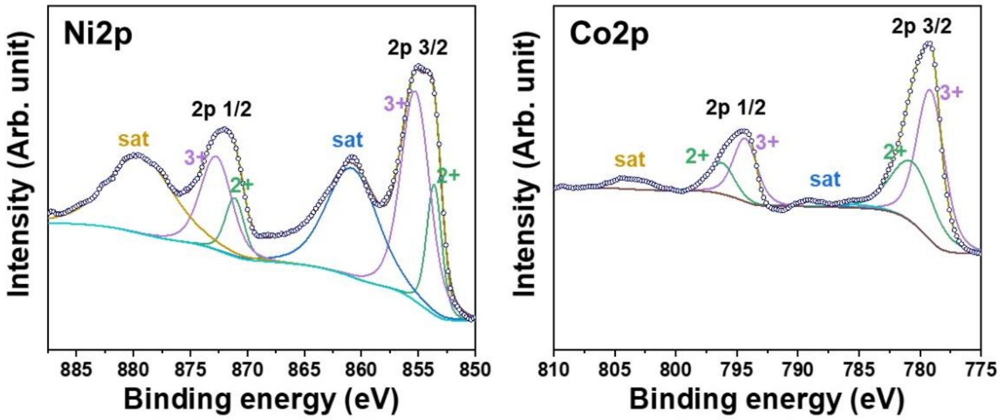

Figure S5. The XPS spectra of Ni2p and Co2p (NCO13).
